# Therapeutic potential of escin and its nanoparticle formulations in cancer -a systematic review and meta-analysis of preclinical study

**DOI:** 10.3389/fphar.2026.1988783

**Published:** 2026-10-30

**Authors:** Mao Xu, Yinju Cui, Dan Li, Ye Cheng, Haiyan Zhang, Jing Li

**Affiliations:** 1 Department of Pharmacy, Hubei Cancer Hospital, Tongji Medical College, Huazhong University of Science and Technology, Wuhan, Hubei, China; 2 Department of Emergency, Union Hospital, Tongji Medical College, Huazhong University of Science and Technology, Wuhan, Hubei, China

**Keywords:** anti-tumor, Escin, meta-analysis, nanoparticle, preclinical study

## Abstract

**Introduction:**

The present study aimed to perform a systematic review and meta-analysis to evaluate the potential therapeutic effects and underlying mechanisms of Escin and its nanoparticle formulations in cancer based on preclinical studies.

**Methods:**

English databases including PubMed, Web of Science and Embase were used to search all relevant articles from their inception to July 13, 2026. The risk of bias of the included studies was assessed using the SYRCLE animal experiment risk assessment tool, while Review Manager (RevMan) software (Version 5.4) and Stata SE 12 software were employed for all statistical computations. Egger’s test was used to assess potential publication bias.

**Results:**

The initial search produced a total of 279 potentially pertinent studies, and 34 studies met the inclusion criteria. The meta-analysis findings demonstrated Escin significantly inhibited cancer cell growth in vitro. Compared with the control group, Escin substantively reduced cell viability (SMD = ‐4.73, 95% CI = [‐5.81, ‐3.65]), induced cell cycle arrest (SMD = -7.29, 95% CI = [‐10.45, ‐4.14]), increased cell apoptosis (SMD =4.88, 95% CI = [3.41, 6.36]), and inhibited cell colony formation (SMD = -8.15, 95% CI = [‐14.04, ‐2.26]), cell adhesion (SMD = ‐11.17, 95% CI = [‐17.80, ‐4.55]), and migration (SMD = ‐3.19, 95% CI = [‐4.80, ‐1.57]). In tumor‐bearing animals, Escin treatment reduced tumor weight (SMD =‐2.49, 95% CI = [‐3.27, ‐1.70]), tumor volume (SMD = ‐2.05, 95% CI = [‐2.87, ‐1.23]), and tumor number (SMD = ‐1.83, 95% CI = [‐2.84, ‐0.82]). Besides, Escin nanoformulations also significantly reduced cell viability (SMD = ‐3.12, 95% CI = [‐5.15, ‐1.09]).

**Discussion:**

Overall, this meta-analysis provided quantitative preclinical evidence supporting the potential anti‐tumor effects of Escin and its nanoformulations. However, given the heterogeneity and limited number of included studies, especially for nanoformulations, these findings should be interpreted cautiously. Further well‐designed preclinical studies are warranted to facilitate clinical translation.

**Systematic Review Registration:**

https://www.crd.york.ac.uk/PROSPERO/.

## 1 Introduction

Cancer is a global public health problem. It is the second-leading cause of death overall and the leading cause among people younger than 85 years (Siegel et al., 2025). Currently, cancer diagnosis and treatment rely on conventional treatments including surgery, radiotherapy and chemotherapy, alongside advanced strategies and agents such as gene therapy, stem-cell therapy, targeted therapy, photodynamic therapy, nanomedicine and precision medicine (Kaur et al., 2023). Nevertheless, major challenges such as drug-resistant recurrence and systemic toxicity remain unresolved despite therapeutic progress (Zhou et al., 2023). Accordingly, there is an urgent need to develop more effective and safer anti-cancer agents or adjunctive strategies.

Escin is a naturally triterpenoid saponin mixture extracted from the seeds of *Aesculus hippocastanum*. It was initially reported to have antiedematous, anti-inflammatory and venotonic properties (Gallelli, 2019), Based on the position of acetyl substitution, Escin can be divided into three components including β-aescin, crypto-aescin, and α-aescin (with α-aescin being a mixture of β-aescin and crypto-aescin) (Wang et al., 2023). In particular, sodium aescinate, the sodium salt of natural Escin prepared by reaction with sodium hydroxide, has been shown to be effective in treating brain edema and swelling caused by trauma or surgery (Zhang et al., 2025). Recently, accumulating evidences have shown that Escin is a promising candidate anti-tumor chemotherapeutic agent, and its underlying mechanisms are being progressively unraveled (Li et al., 2023). A prior review has systematically summarized Escin’s chemical properties and bioavailability, with particular emphasis on its inhibitory activities against various cancer types (Fazliev et al., 2023). Multiple recent studies have further demonstrated that monotherapy with Escin can effectively suppress tumor growth in breast and colon cancers (Ibrahim et al., 2023; Park et al., 2022). Moreover, Escin triggerd ferroptosis and exerted synergistic anti-cancer effects when combined with cisplatin (Li et al., 2023), it confered chemosensitization in human pancreatic cancer cells through suppression of the Nuclear Factor-kappaB (NF-κB) signaling cascade (Rimmon et al., 2013), and potentiated the therapeutic response to PD-1 immune checkpoint inhibitors in hepatocellular carcinoma (Yuan et al., 2025). Collectively, these findings underscore the promising anti-cancer potential of Escin and its value for cancer therapeutic development.

Although anti-cancer activity has been confirmed, the clinical transformation of many natural active ingredients is generally restricted by bottlenecks such as poor water solubility, low bioavailability and rapid metabolic clearance in the body (Wang et al., 2022). However, nanomedicines are expected to fundamentally break through these obstacles (Mao et al., 2022). It is worth noting that the feasibility of Escin nanoformulations has been preliminarily verified. For example, Escin-loaded chitosan nanoparticles displayed anti-arthritic activity in Wistar rats (Riaz et al., 2026). More importantly, the MgO-Chitosan-Pluronic F127-Escin (MCsPFE) nanoparticles augmented the ROS levels in breast cells and altered the Δψm, leading to the initiation of the intrinsic apoptotic cell death pathway for anti-cancer treatment (Mickymaray et al., 2023). Collectively, these findings strongly suggest that Escin-based nanocarrier systems hold great promise for cancer therapy, which need further investigation into their efficacy and mechanisms in relevant tumor models.

Despite extensive evidence from cellular and animal studies confirming the anti-tumor activity of Escin, its clinical translation remains hindered by drawbacks including poor water-solubility and limited bioavailability (Franiczek et al., 2015). Although several studies have demonstrated the feasibility of Escin-based nanocarrier systems, the overall magnitude of pre-clinical anti-tumor effects of both free Escin and Escin nanoformulations has not been quantitatively synthesized. Moreover, there is no formal quality evaluation of existing animal studies investigating Escin-related anti‐tumor effects, and antitumor mechanistic information remains scattered across individual publications. To address these gaps, the present systematic review and meta-analysis of preclinical studies was designed to quantitatively evaluate the anti-tumor effects of free Escin and Escin nanoformulations and assess the risk of bias of included animal studies. Furthermore, we summarized the anti-tumor molecular mechanisms and highlighted key limitations to offer insights for future translational research.

## 2 Methods

### 2.1 Guidance and protocol

The meta-analysis was conducted in accordance with the Preferred Reporting Items for Systematic Reviews and Meta-Analyses (PRISMA) guidelines. The study has been registered on the International Prospective Register of Systematic Reviews (PROSPERO) with the identification number CRD420261376092.

### 2.2 Search strategy

To obtain comprehensive studies that may be related to the treatment of cancer with Escin or its nanoformulations, we retrieved three English databases of PubMed, Web of Science and Embase from their inception to 13 July 2026. The MeSH and free-text terms were suitably modified for each database to ensure comprehensive and relevant search results. The search terms were “Escin”, “Sodium Aescinate”, “β-Escin”, “β-Aescine”, “Aescine”, “tumor”, “cancer”, “carcinoma”, “neoplasm”, “glioma”, “glioblastoma” and “neuroblastoma”. In addition, the relevant reviews, as well as the references in the reviews, were studied to search for other eligible studies.

### 2.3 Study selection

The criteria for inclusion in this study were according to the PICOS principles (population, intervention, comparison, outcome, and study design).

Subjects (P): *in vivo* and *in vitro* cancer models.

Intervention (I): single Escin or its nanoformulation treatment for cancer.

Comparison (C): an equivalent amount of a placebo or saline controlled.

Outcome (O): cell viability, cell cycle, cell apoptosis, cell clone, cell adhesion, invasion, migration, cell proliferation number, tumor volume, tumor number, tumor weight, alanine aminotransferase (ALT), and aspartate aminotransferase (AST).

Study design (S): *in vitro* experiments, different types of tumor cells treated with or without Escin and its nanoparticles; *in vivo* experiments, animal inoculated different types of tumor cells and treated with or without Escin and its nanoparticles.

The criteria for excluding studies from selection include: (1) repeated studies; (2) reviews; (3) conference abstracts; (4) case reports; (5) meta-analyses; (6) cohort studies; (7) studies lacking data on the quantification of outcome measures; (9) studies where the full text is not available.

### 2.4 Data extraction

Endnote X9 was used to identify and remove duplicate or inappropriate studies. To ensure accuracy, two reviewers independently screened the titles, abstracts and full texts to identify studies eligible for inclusion according to outlined inclusion and exclusion criteria. In case of disagreement, the final decision was made by the third-party reviewer. When a single publication reported multiple experiments using different cell lines or different cancer models that were independent experimental systems, each experiment was treated as a separate study and ^a, b, c, d^ was used to distinguish them. The extracted information from the literature included first author’s name, publication year, detailed information about the animals used (number, strains, gender and age/weight), cell lines, detail of Escin invention (formulation, dose, duration, and administration method) and pertinent outcomes. The mean and standard deviation (*SD*) for the graphical results were extracted using GetData Graph Digitizer 2.26.

### 2.5 Methodological quality and assessment of studies

To assess the risk of bias in the studies, two reviewers independently assessed the risk of bias using the SYRCLE’s risk of bias tool. In case of any ambiguity in the screening results, consult with a third-party negotiation to arrive at a consensus conclusion. The bias risks assessment results were classified into three categories: “low”, “high” and “uncertain”.

### 2.6 Data analysis

Data analysis was performed using Review Manager (RevMan) software (Version 5.4) and Stata SE 12 Software. Given that the outcomes indices in this research were continuous variables, effect sizes were estimated using standardized mean differences (SMD) along with their 95% confidence intervals (CIs). Statistical significance between the intervention and control groups was defined as a *P*-value < 0.05. Heterogeneity across studies was assessed using the *I* ^*2*^ statistic. An *I* ^*2*^ value ≤ 50% was considered indicative of homogeneity, whereas value > 50% was interpreted as evidence of substantial between-study heterogeneity. In the latter case, a random-effects model was employed to estimate pooled effect sizes. Sub-group analysis was applied to investigate possible determinants of heterogeneity among the included studies. In addition, *Egger’s* test was conducted to evaluate publication bias.

## 3 Results

According to the search strategy, a total of 279 records were retrieved from three databases. Following the removal of duplicates, 123 records remained. Subsequent to a screening process of titles and abstracts, an additional 58 irrelevant records were excluded. Finally, 34 records were selected for inclusion in this meta-analysis after a meticulous full-text review. The retrieval process is depicted in Figure 1.

**FIGURE 1:**
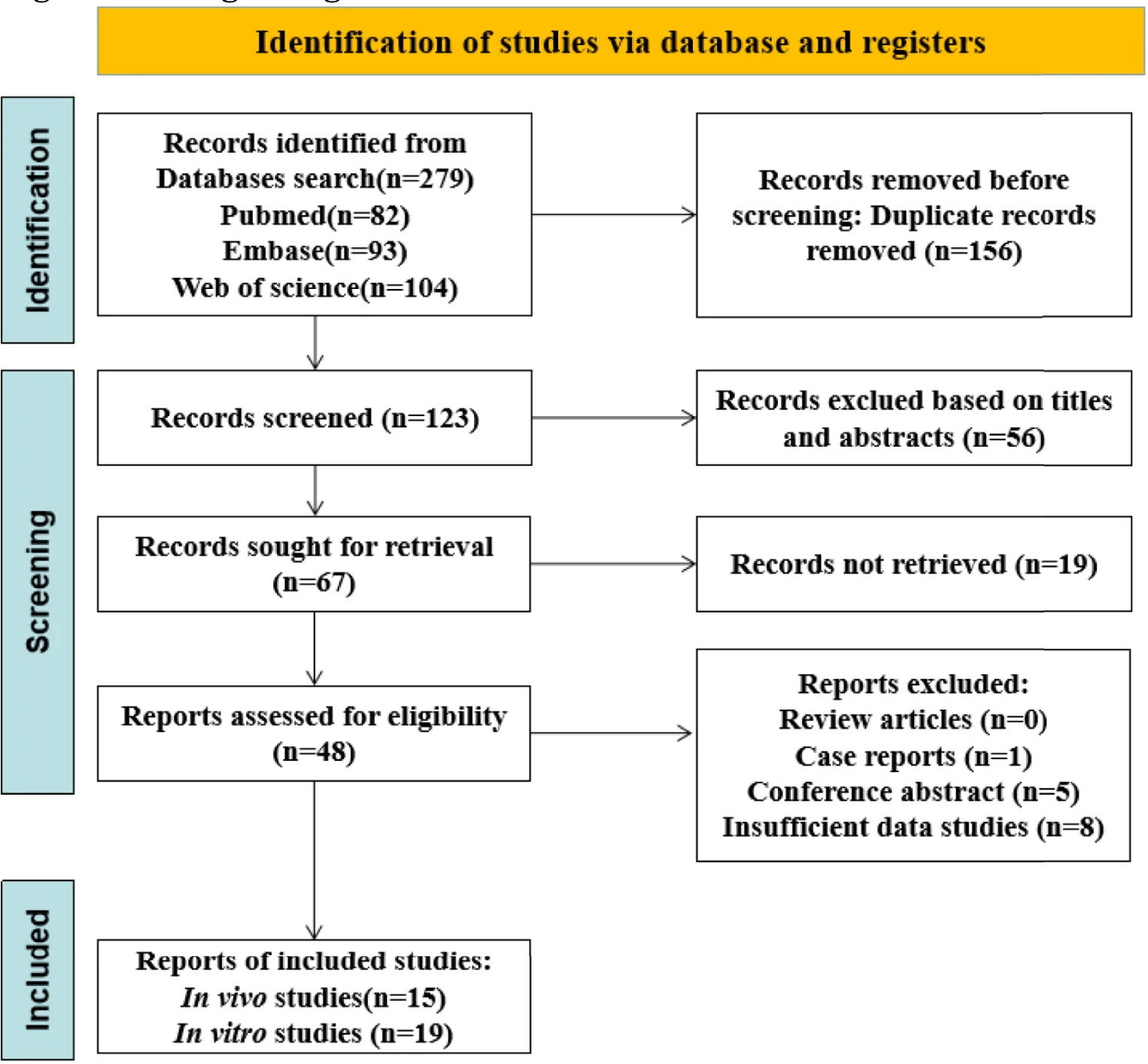
Flow diagram for identification of relevant studies (Light blue represents the process).

### 3.1 Characteristics of the included studies

Table 1 lists the detailed characteristics of the included study *in vitro*, tumor cell lines used for evaluation included HepG2 (*n* = 3), A549 (*n* = 4), MCF-7 (*n* = 6), MDA-MB-231 (*n* = 4), SMA (*n* = 2), AGS (*n* = 2) and other representative cells of tumor types. Additionally, a total of 15 articles (16 studies) focusing eight types of cancer, including ovarian cancer (*n* = 3), lung cancer (*n* = 1), breast cancer (*n* = 1), prostate cancer (*n* = 2), liver cancer (*n* = 2), colorectal cancer (*n* = 1), osteosarcoma (*n* = 1), were included *in vivo* studies (Table 2). Six strains (BALB/c nude mice, *n* = 10; BALB/c mice, *n* = 1; C57 B l/6, *n* = 1; HSD nude mice, *n* = 1; KM mice, *n* = 1; AJ mice, *n* = 1) were used. Female (*n* = 10), male (*n* = 4), and unclear (*n* = 2) were reported across the included studies. The sample sizes of experimental animals ranged from 3 to 18. Doses of drug ranged from 1 mg/kg to 10 mg/kg, and were administered through intraperitoneally injected (*i.p.*, *n* = 10), intravenously injected (*i.v.*, *n* = 1), or intragastric (i.g., *n* = 5), with treatment durations ranging from 7 days to 119 days. Moreover, a total of three articles focused on Escin nanoparticles were included, and the details are provided in Supplementary Table 1.

**TABLE 1:** Characteristics of studies on Escin for cancer therapy.

Country	Cell strains	Cancer	Treatment (dose; route; frequency)	Outcomes	Refs
China	T24, RT-4, J82	Bladder cancer	1.5, 3 mg/kg,i.p., 2 days/week	1, 2, 9	(Cheng et al., 2018)
Turkey	C6, A549	Glioma, Lung cancer	2 mg/mL	1, 2, 3	(Çiftçi et al., 2015)
Saudi Arabia	MOLT-4	Leukemia	1, 1.5, 2 mg/mL	1	(Elderdery et al., 2022)
France	U87-MG, SK-N-SH	Glioblastoma	10 μM	1, 3	(Harford-Wright et al., 2016)
China	HepG2, Hep3B	Liver cancer	40 μM	1, 3, 4	(Hou et al., 2019)
China	A549	Lung cancer	5, 10, 20, 40 mg/mL	1	(Ji et al., 2011)
United States of America	HeyA8, SNU-119, Kuramochi, Ovcar4, Ovcar5	Ovarian cancer	1, 2 mg/kg, i.g., daily	6, 8, 10, 11	(Kenny et al., 2021)
Korea	B16F10, SK-MEL5	Melanoma	2, 6, 20 μM	5	(Kwak et al., 2018)
Korea	AGS	Gastric cancer	2, 4 μM	6, 7	(Lee et al., 2014)
China	MDA-MB-231, MCF7	Breast cancer	2 mg/kg, i.p., daily	1, 3, 9	(Li et al., 2020)
China	A549	Lung cancer	10 μg/kg PTX, i.v., per 2 days	1, 2, 3, 8, 9, 11	(Li et al., 2020)
France	MCF7	Breast cancer	20 μg/mL	1, 3, 4	(Mazrouei et al., 2019)
Saudi Arabia	MDA-MB-231	Breast cancer	10 μg/mL	1, 3	(Mickymaray et al., 2023)
Slovak	Jurkat, CEM, HeLa, MCF7	Melanoma, Cervical, Breast cancer	7.8, 15.6 μM	1, 2, 3, 4	(Mojzisova et al., 2016)
Australia	BE2-C, MCF7, SMA, MiaPaCa-2	Neuroblastoma, Breast, Pancreatic, Glioblastoma	25, 50 μM	1	(Nguyen et al., 2017a)
Australia	BE2-C, MCF7, SMA, MiaPaCa-2	Neuroblastoma, Breast, Pancreatic, Glioblastoma	​	1	(Nguyen et al., 2017b)
Japan	BxPC-3, AsPC-1, SW1990	Pancreatic cancer	20 μM	1	(Omi et al., 2021)
India	A549	Lung cancer	12.5 μM	1, 5, 6	(Paneerselvam and Ganapasam, 2020)
Korea	SKBR3, BT474, JIMT-1, MDAMB-453, MDA-MD-231	Breast cancer	4 mg/kg, i.p., 5 days per week	1, 2, 3, 9, 11	(Park et al., 2022)
Canada	HT-29, HCT-116	Colon cancer	0.025% or 0.05%, i.g., daily	2.3	(Patiolla et al., 2006)
Canada	NCI-H460	Lung cancer	500 ppm, i.g., daily	10	(Patlolla et al., 2013)
Korea	PC-3, DU-145, PNT-2	Prostate cancer	1.4, 2.8 mg/kg, i.p., daily	1, 2, 3, 9, 11	(Piao et al., 2014)
Israel	Panc-1, COLO 357, MIA-Paca	Pancreatic cancer	10, 20, 30 μM	2	(Rimmon et al., 2013)
Turkey	CHL-1	Melanoma	3.5 μg/mL	1	(Vejselova Sezer, 2024)
Singapore	HepG2, PLC/PRF5, HUH-7	Liver cancer	30 μM	3	(Tan et al., 2010)
China	MDA-MB-231, MCF7	Breast cancer	2.4 mg/kg, i.p., 3 days per week	6, 7	(Wang et al., 2016)
China	H22	Liver cancer	1.4, 2.8 mg/kg, i.p., 2 days per week	11	(Wang et al., 2012a)
China	BxPC-3, PANC-1, CFPAC-1, SW-1990	Pancreatic cancer	2 mg/kg, i.p., daily	1, 2, 3, 9	(Wang et al., 2012b)
China	HCT116, HCT8	Colorectal cancer	2 mg/kg, i.p., daily	1, 3, 4, 11	(Wang et al., 2018)
China	786-O, Caki-1	Kidney cancer	5, 10, 20 μg/mL	1, 2, 3	(Yuan et al., 2017)
China	Jurkat	Leukemia	12.5, 25, 50 μg/mL	1, 2	(Zhang et al., 2011)
China	H22, HepG2	Liver cancer	1.4,2.8 mg/kg, i.p., daily	2, 11	(Zhou et al., 2009)
China	MNNG/HOS, Saos-2, MG-63, U2-OS	Osteosarcoma	1.4,2.8 mg/kg, i.p., daily	3	(Zhu et al., 2017)
China	MNNG/HOS, Saos-2, MG-63, U2-OS	Osteosarcoma	5,10 mg/kg, i.g., daily	3, 9, 11	(Zhu et al., 2018)

1. Cell viability, 2. Cell cycle, 3. Cell apoptosis, 4. Cell clone, 5. Cell adhesion, 6. Invasion, 7. Migration, 8. Cell proliferation number, 9. Tumor volume, 10. Tumor number, 11. Tumor weight.

**TABLE 2:** Characteristics of *in vivo* studies on Escin for cancer therapy.

Animal species (sex/age or weight)	Sample size (n) treatment/control	Cancer	Treatment (dose, route, frequency)	Follow-up (days)	Outcomes	Refs
BALB/c nude mice (female, 6 weeks, 20–25 g)	5/5	Bladder cancer	1.5 or 3 mg/kg, i.p., 2 days per week	25	1, 2, 9	China (Cheng et al., 2018)
1. C57Bl/6 (female, 5–6 weeks, 20 g) 2. HSD nude mice (female, 5–6 weeks, 20 g)	1. C57Bl6/6 (5/5) 2. HSD nude (10/10)	Ovarian cancer	1, 2 mg/kg, i.g., daily	45	6, 8, 10, 11	United States of America (Kenny et al., 2021)
BALB/c nude mice (female, 4-week-old)	3/3	Breast cancer	2 mg/kg, i.p., daily	14	1, 3, 9	China (Li et al., 2023)
BALB/c nude mice (female, 6-week-old)	5/5	Lung cancer	10 μg/kg PTX, i.v., per 2 days	14	1, 2, 3, 8, 9, 11	China (Li et al., 2020)
BALB/c nude mice (female, 5-week-old)	8/8	Breast cancer	4 mg/kg, i.p., 5 days per week	28	1, 2, 3, 9, 11	Korea (Park et al., 2022)
F344 rats (male, six-week-old)	18/18	Colon cancer	0.025% or 0.05%, i.g., daily	21	2, 3	Canada (Patiolla et al., 2006)
A/J mice (female, 6-week-old)	10/5	Lung cancer	500 ppm, i.g., daily	119	10	Canada (Patlolla et al., 2013)
BALB/c nude mice (female, 5-week-old)	5/5	Prostate cancer	1.4, 2.8 mg/kg, i.p., daily	35	1, 2, 3, 9, 11	Korea (Piao et al., 2014)
BALB/c nude mice (female, 4–6 week-old)	​	Breast cancer	2, 4 mg/kg, i.p., 3 per days week	35	6.7	China (Wang et al., 2016)
BALB/c mice	15/15	Liver cancer	1.4, 2.8 mg/kg, i.p., 2 days per week	14	11	China (Wang et al., 2012a)
BALB/c nude mice (male, 4–6 week-old)	5/5	Pancreatic cancer	2 mg/kg, i.p., daily	21	1, 2, 3, 9	China (Wang et al., 2012b)
BALB/c nude mice (female, 6 week-old)	5/5	Colorectal cancer	2 mg/kg, i.p., daily	14	1, 3, 4, 11	China (Wang et al., 2018)
KM mice (female, 6–8 week-old, 18–20 g)	6/6	Liver cancer	1.4, 2.8 mg/kg, i.p., daily	7	2, 11	China (Zhou et al., 2009)
BALB/c nude mice (male, 4 weeks old)	5/5	Osteosarcoma	1.4, 2.8 mg/kg, i.p., daily	21	3	China (Zhu et al., 2017)
BALB/c nude mice (male, 6-week-old)	6/6	Osteosarcoma	5, 10 mg/kg, i.g., daily	21	3, 9, 11	China (Zhu et al., 2018)

1. Cell viability, 2. Cell cycle, 3. Cell apoptosis, 4. Cell clone, 5. Cell adhesion, 6. Invasion, 7. Migration, 8. Cell proliferation number, 9. Tumor volume, 10. Tumor number, 11. Tumor weight.

### 3.2 Risk of bias

A comprehensive quality assessment of the 15 articles included was conducted using the SYRCLE tool (Figure 2; Supplementary Figure 1). Of these, eight studies explicitly reported the use of randomization although none described the specific methods applied, while the remaining studies did not indicate whether randomization had been performed. 14 studies provided detailed information on the animals’ species, sex, and age or weight. In contrast, the last one only reported the species. All animals were randomly assigned to housing conditions. However, none of the studies reported the use of blinding, nor did they specify whether outcome assessment was performed in a randomized manner. Most studies included all animals in the final analysis, except 2 studies, which had no animal experiment outcome indicators. Nonetheless, the information provided in that article was insufficient to determine whether the data were missing. Overall, based on these, the above studies were considered suitable for inclusion in this meta-analysis.

**FIGURE 2:**
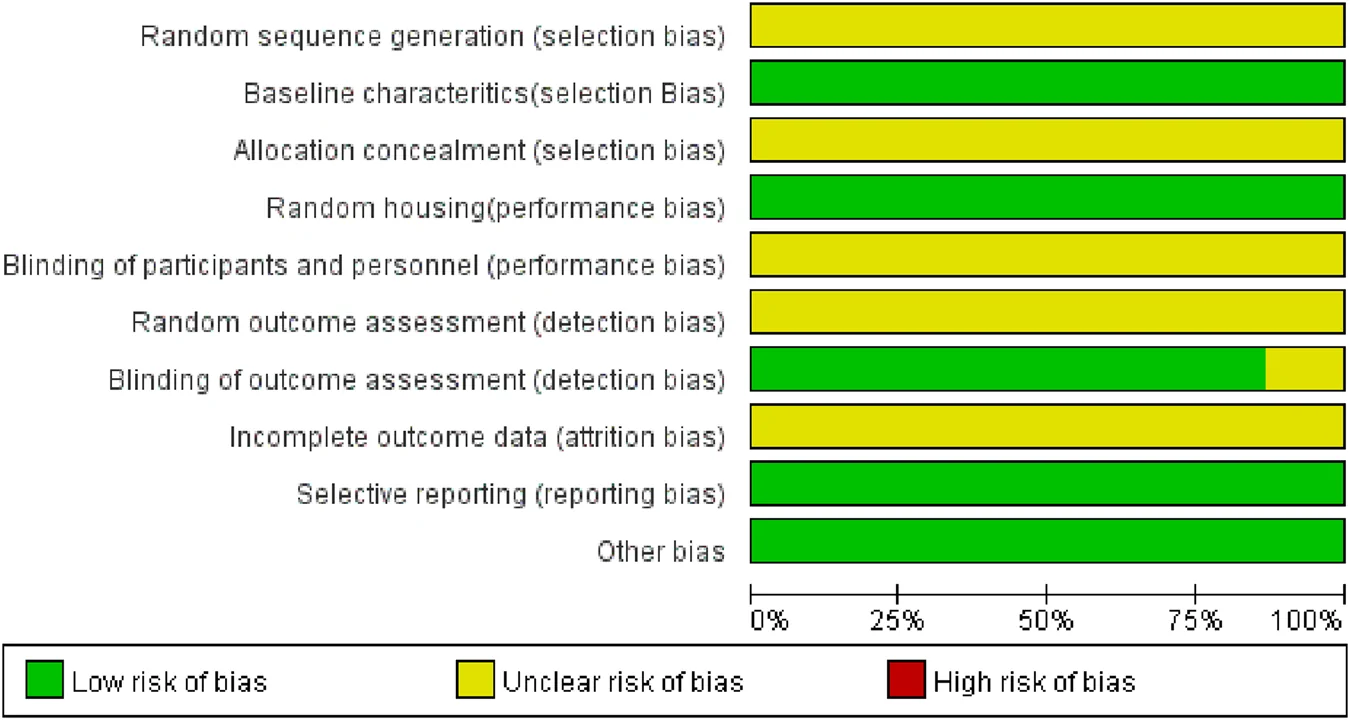
The risk of bias graph of included studies using SYRCLE’s risk of bias tool for animal studies.

### 3.3 The effect of Escin on cancer cell’s biology function *in vitro*

Twenty-two articles including 35 studies evaluated the Escin’s effect on cancer cell viability at the cellular level. As shown in Figure 3, Escin at the concentrations of 1–40 μM exerted substantial reduction in cancer cell viability (SMD = −4.72, 95% CI = [-5.79, −3.64], *P* < 0.00001; *I* ^*2*^ = 39%). Given that uncontrolled proliferation is a significant characteristic of malignant cells, the magnitude of this viability reduction provides compelling evidence for the concentration-dependent anti-tumor activity of Escin. To further eluciate the mechanistic basis of this effect, Escin’s impact on cell cycle regulation and apoptotic induction were assessed. Results showed Escin could stimulate the tumor cells to enter a G1 phase of the cell cycle (SMD = −9.72, 95% CI = [-15.67, −3.77], *P* = 0.001; *I* ^*2*^ = 72%) and significantly reduce the proportion of G1/G0 phase cells (SMD = −5.22, 95% CI = [-7.63, −2.81], *P* < 0.0001; *I* ^*2*^ = 0%). The aggregated effect on cell cycle was highly significant (SMD = −7.29, 95% CI = [-10.45, −4.14], *P* < 0.00001; *I* ^*2*^ = 62%), with no evidence of subgroup heterogeneity (*P* = 0.17) (Figure 4A). Concurrently, Escin markedly promoted apoptosis with a pooled effect size of 4.88 (95% CI = [3.41, 6.36], *P* < 0.00001; *I* ^*2*^ = 46%) (Figure 4B). These results collectively suggested that Escin exerted cytotoxic effects at least in part through the dual mechanisms of cell-cycle perturbation and apoptosis enhancement.

**FIGURE 3:**
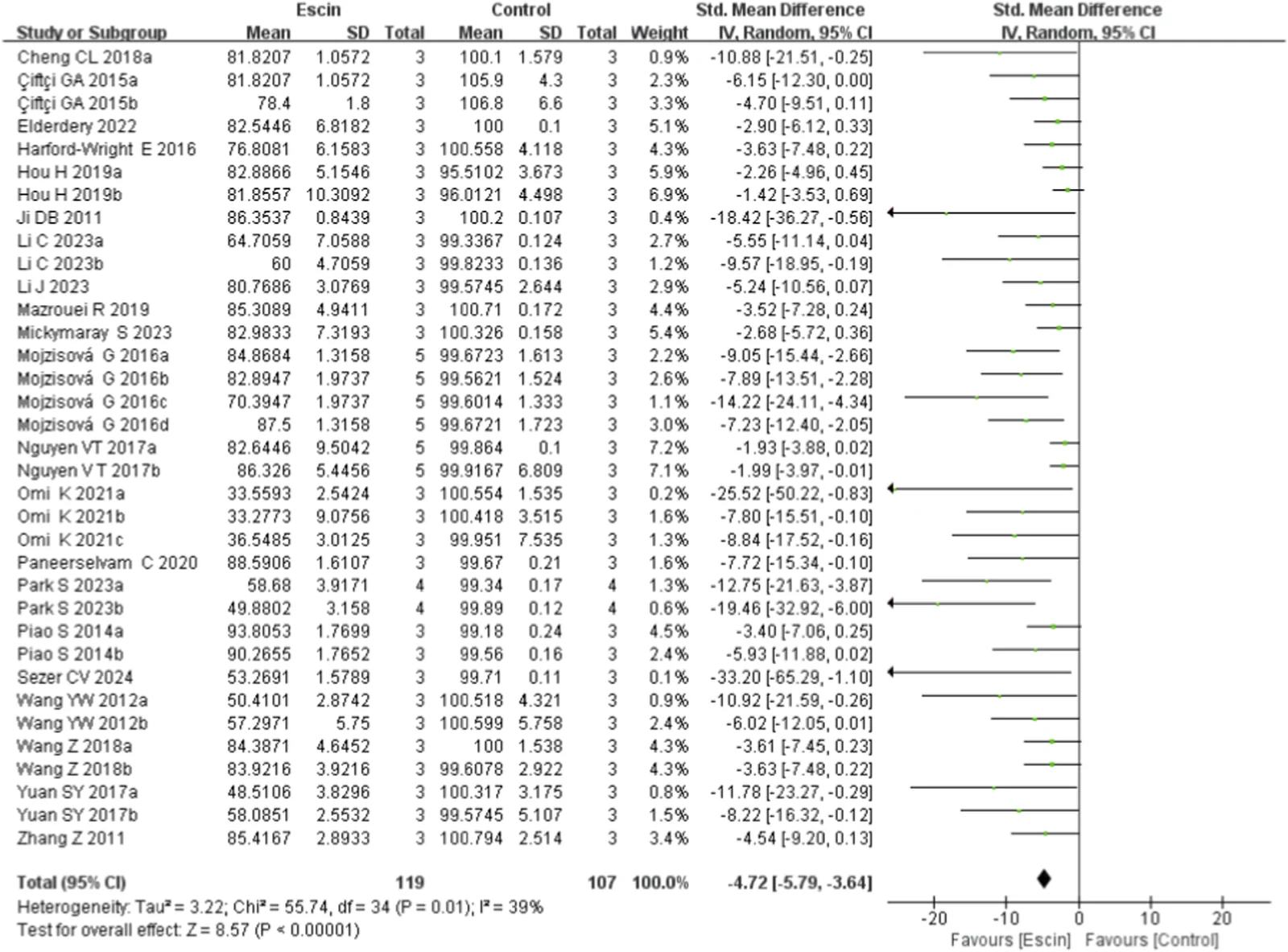
Forest plot for the effects of Escin on the cell viability compared with the model control group. ^a, b, c, d^ was used to distinguish the different cell lines used in the same article.

**FIGURE 4:**
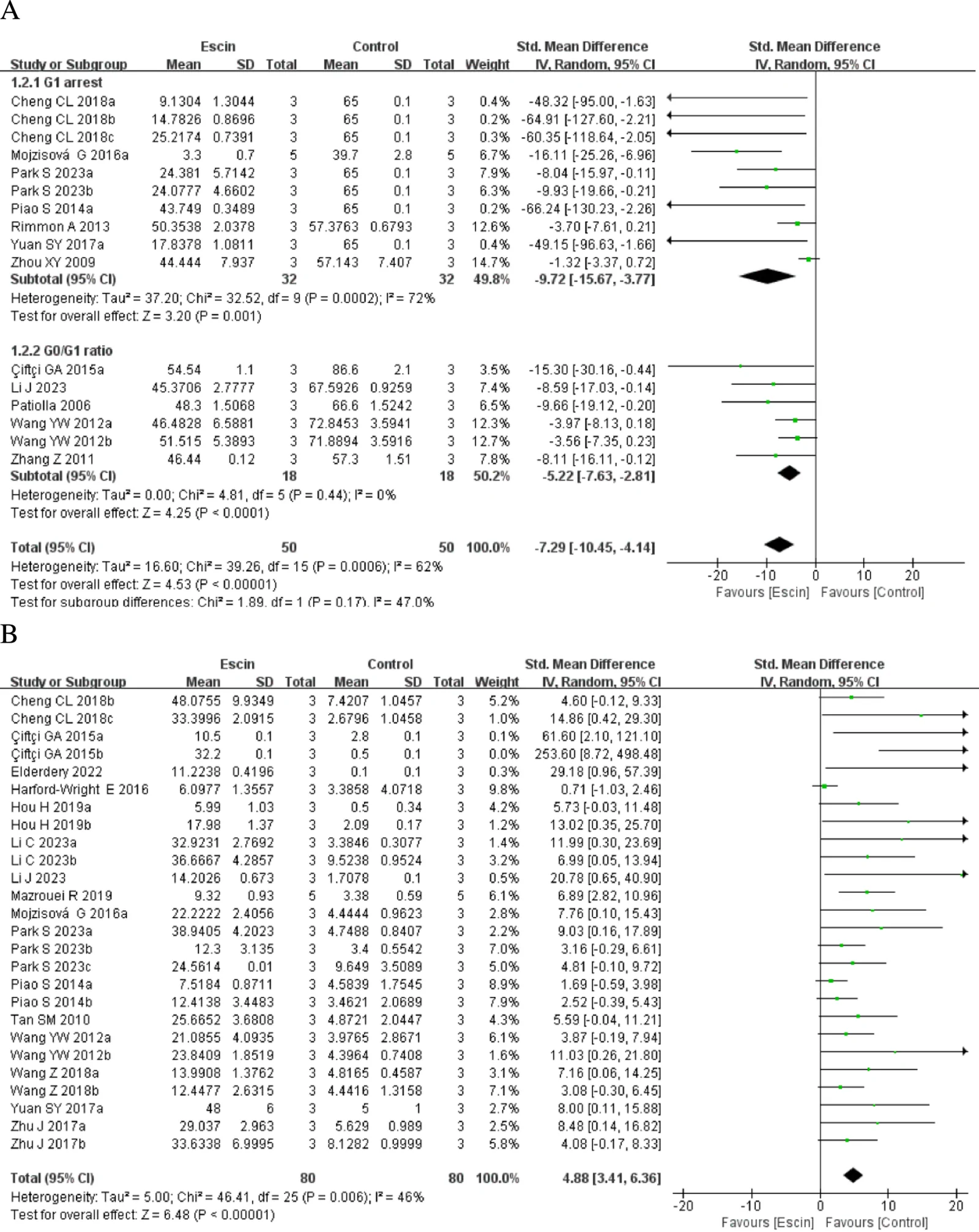
Forest plot for the effects of Escin on the **(A)** cell cycle arrest (G1 and G0/G1 ratio) and **(B)** cell apoptosis compared with the model control group. ^a, b, c^ was used to distinguish the different cell lines used in the same article.

Besides, Escin also impaired other critical determinants of tumor malignancy, namely, cell clone, cell adhesion and migration (Supplementary Figure 2). Quantitative synthesis revealed that Escin significantly reduced cell clone (SMD = −8.15, 95% CI = [-14.04, −2.26], *P* = 0.007; *I* ^*2*^ = 62%), cell adhesion (SMD = −11.17, 95% CI = [-17.80, −4.55], *P* = 0.0009; *I* ^*2*^ = 0%) and migration (SMD = −3.19, 95% CI = [-4.80, −1.57], *P* = 0.001; *I* ^*2*^ = 32%).

### 3.4 The effect of Escin on tumor weight and volume *in vivo*

A total of eight articles (11 studies) reported tumor weight, seven articles (8 studies) reported tumor volume, and two articles (4 studies) reported tumor number between Escin and the untreated group in animal cancer models, respectively. Pooled analysis indicated that Escin administration resulted in substantial reductions in tumor weight (SMD = −2.49, 95% CI = [-3.27, −1.70], *P* < 0.00001; *I* ^*2*^ = 61%), tumor volume (SMD = −2.05, 95% CI = [-2.87, −1.23], *P* < 0.00001; *I* ^*2*^ = 29%), and tumor number (SMD = −1.83, 95% CI = [-2.84, −0.82], *P* < 0.0004; *I* ^*2*^ = 62%) (Figure 5).

**FIGURE 5:**
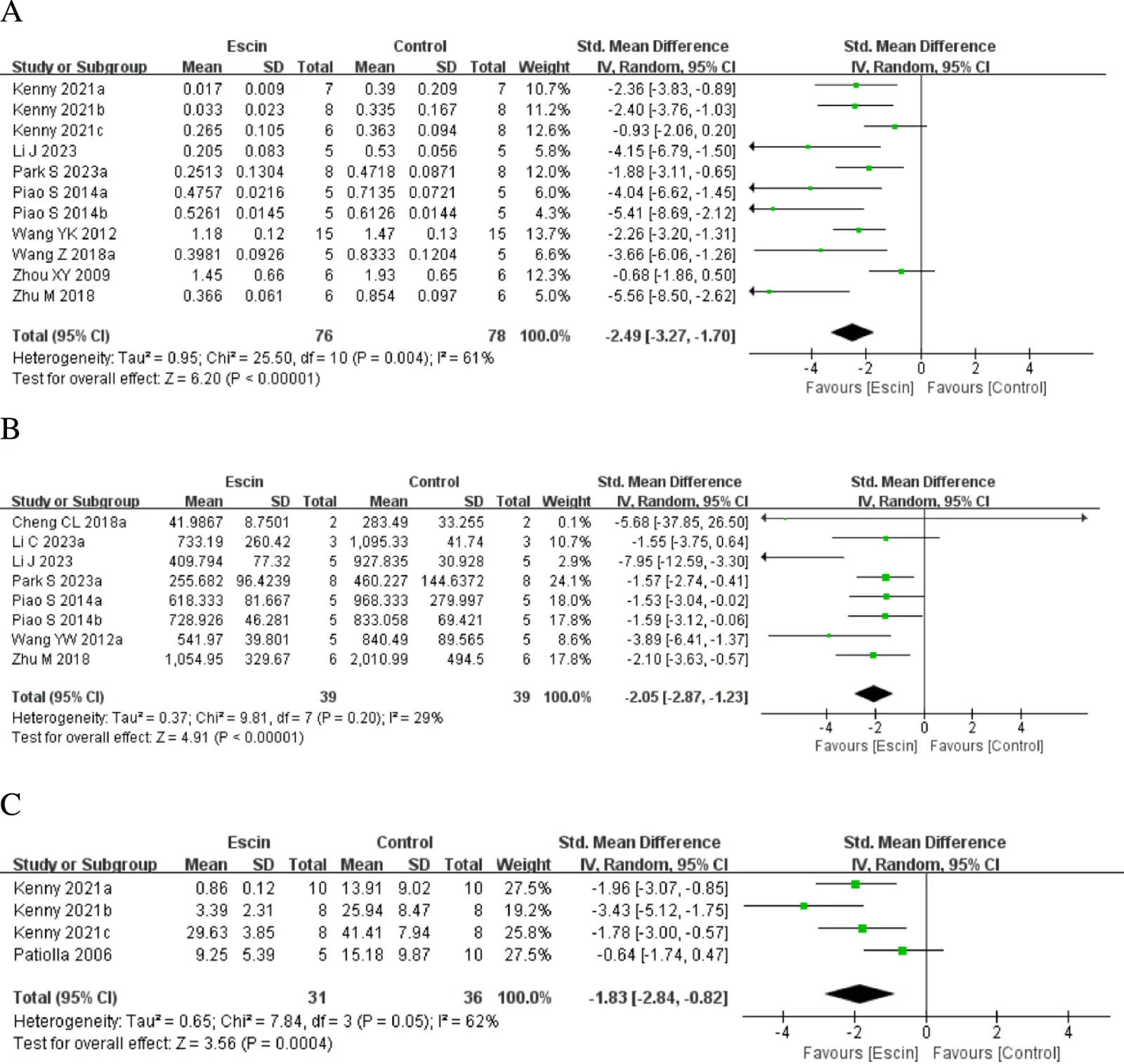
Forest plot for the anti-tumor effects of Escin on Rodents compared with the model control group. **(A)** tumor weight. **(B)** tumor volume, **(C)** tumor number, ^a, b, c^ was used to distinguish the different tumor types used in the same article.

Given the substantial heterogeneity observed across the included studies, subgroup analyses were performed to explore its potential sources. Some prespecified confounding factors including animal strains, gender, route of administration, number of animals per group, and type of tumor were examined. For tumor weight, subgroup analysis showed that animal strains (*I* ^*2*^ = 74.8%), animal gender (*I* ^*2*^ = 72.6%), animal numbers (*I* ^*2*^ = 87.6%), and type of tumor (*I* ^*2*^ = 5 9.9%) were the significant heterogeneity factors (*P* < 0.05) (Table 3). The results showed that male had a greater effect size (SMD = −3.92, 95% CI = [-5.78, −2.06]) than that of female (SMD = −1.94, 95% CI = [-2.75, −1.14]). Similarly, animal numbers with n ≤ 5 had a greater effect size (SMD = −4.18, 95% CI = [-5.51, −2.84]) than that of *n* > 5 (SMD = −1.93, 95% CI = [-2.70, −1.17]). In terms of animal strains subgroup, significant difference was found for HSD nude (SMD = −2.38, 95% CI = [-3.38, −1.38]) and BALB/c nude (SMD = −3.75, 95% CI = [-5.09, −2.40]). Additionally, subgroup study based on tumor type revealed significant differences in the inhibitory effect of Escin on tumor weight across ovarian cancer, lung cancer, breast cancer, prostate cancer, liver cancer, colorectal cancer, and osteosarcoma. However, some subgroups contained very few studies (e.g., lung cancer: n = 1), and these results should be interpreted cautiously. The largest effect size was observed for prostate cancer (SMD = −4.56, 95% CI = [-6.59, −2.53]), although this estimate was based on a limited number of studies. With regard to the route of administration, i. v. subgroup showed the largest numerical reduction in tumor weight (SMD = −4.15, 95% CI = [-6.79, −1.50]), followed by the i. p. subgroup (SMD = −2.45, 95% CI = [-3.54, −1.36]) and the i. g. subgroup (SMD = −2.39, 95% CI = [-3.78, −0.99]). However, the difference among routes was not statistically significant (*P* = 0.48). For tumor volume, no significant heterogeneity was observed in the subgroup analysis (Table 4).

**TABLE 3:** Subgroup analysis for tumor weight.

Study characteristics	Participants			Test for heterogeneity			Test for effect		Subgroup
	Studies	Escin	Con	*I* ^*2*^ (%)	*Chi* ^*2*^ test	*P*-value	SMD [95% CI]	*P*-value	Statistics, *P*-value, *I* ^*2*^
Strains									
HSD nude	2	15	15	0	0	*P* = 0.97	−2.38 [-3.38, −1.38]	*P* < 0.00001	7.95, *P* = 0.02, 74.8%
BALB/c nude	6	34	34	48	9.63	*P* = 0.09	−3.75 [-5.09, −2.40]	*P* < 0.00001	​
others	3	27	29	62	5.27	*P* = 0.07	−1.34 [-2.35, −0.32]	*P* = 0.10	​
Total	11	76	78	61	25.50	*P* = 0.004	−2.49 [-3.27, −1.70]	*P* < 0.00001	​
Gender									
Female	7	45	47	51	12.34	*P* = 0.05	−1.94 [-2.75, −1.14]	*P* < 0.00001	3.65, *P* = 0.004, 72.6%
Male	4	31	31	62	7.91	*P* = 0.05	−3.92 [-5.78, −2.06]	*P* < 0.0001	​
Total	11	76	78	61	25.50	*P* = 0.004	−2.49 [-3.27, −1.70]	*P* < 0.00001	​
Administration									
i.g	4	27	29	69	9.60	*P* = 0.02	−2.39 [-3.78, −0.99]	*P* = 0.0008	1.48, *P* = 0.48, 0%
i.p	6	44	44	63	13.46	*P* = 0.02	−2.45 [-3.54, −1.36]	*P* < 0.00001	​
i.v	1	5	5	-	-	​	−4.15 [-6.79, −1.50]	*P* = 0.002	​
Total	11	76	78	52	17.18	*P* = 0.03	−2.49 [-3.27, −1.70]	*P* < 0.00001	​
Number									
n > 5	7	56	58	58	14.23	*P* = 0.03	−1.93 [-2.70, −1.17]	*P* < 0.00001	8.1, *P* = 0.004, 87.6%
n ≤ 5	4	20	20	0	0.73	*P* = 0.87	−4.18 [-5.51, −2.84]	*P* < 0.00001	​
Total	11	76	78	61	25.50	*P* = 0.004	−2.49 [-3.27, −1.70]	*P* < 0.00001	​
Type of tumor									
Ovarian cancer	3	21	23	43	3.53	*P* = 0.17	−1.81 [-2.82, −0.80]	*P* = 0.0004	14.97, *P* = 0.02, 59.9%
Lung cancer	1	5	5	-	-	-	−4.15 [-6.79, −1.50]	*P* = 0.002	​
Breast cancer	1	8	8	-	-	-	−1.88 [-3.11, −0.65]	*P* = 0.003	​
Prostate cancer	2	10	10	0	0.41	*P* = 0.52	−4.56 [-6.59, −2.53]	*P* < 0.0001	​
Liver cancer	2	30	21	49	1.96	*P* = 0.16	−1.78 [-2.76, −0.80]	*P* = 0.0004	​
Colorectal cancer	1	5	5	-	-	-	−3.66 [-6.06, −1.26]	*P* = 0.003	​
Osteosarcoma	1	6	6	-	-	-	−5.56 [-8.50, −2.62]	*P* = 0.0002	​
Total	​	​	​	​	​	​	−2.49 [-3.21, −1.77]	*P* < 0.00001	​

**TABLE 4:** Subgroup analysis for tumor volume.

Study characteristics	Participants			Test for heterogeneity			Test for effect		Subgroup
	Studies	Escin	Con	*I* ^*2*^ (%)	*Chi* ^*2*^ test	*P*-value	SMD [95% CI]	*P*-value	Statistics, *P*-value, *I* ^*2*^
Gender									
Female	6	28	28	30	7.14	*P* = 0.21	−1.83 [-2.81, −0.86]	*P* = 0.0002	0.79, *P* = 0.37, 0%
Male	2	11	11	30	1.42	*P* = 0.24	−2.70 [-4.36, −1.04]	*P* = 0.001	​
Total	8	39	39	29	9.81	*P* = 0.20	−2.05 [-2.87, −1.23]	*P* < 0.00001	​
Administration									
i.g	1	6	6	-	-	-	−2.10 [-3.63, −0.57]	*P* = 0.007	9.81, *P* = 0.20, 29%
i.p	6	28	28	0	3.07	*P* = 0.69	−1.75 [-2.47, −1.04]	*P* < 0.00001	​
i.v	1	5	5	-	-	-	−7.95 [-12.59, −3.30]	*P* = 0.0008	​
Total	8	39	39	53	17.18	*P* = 0.03	−2.05 [-2.87, −1.23]	*P* < 0.00001	​
Number									
n > 5	2	14	14	0	0.29	*P* = 0.59	−1.77 [-2.69, −0.84]	*P* < 0.00001	0.63, *P* = 0.43, 0%
n ≤ 5	6	25	25	46	9.28	*P* = 0.10	−2.44 [-3.82, −1.06]	*P* < 0.00001	​
Total	8	76	78	29	9.81	*P* = 0.20	−2.05 [-2.87, −1.23]	*P* < 0.00001	​
Type of tumor									
Bladder cancer	1	2	2	-	-	-	−5.68 [-37.85, −26.50]	*P* = 0.73	9.80, *P* = 0.08, 49.0%
Breast cancer	2	11	11	0	0	*P* = 0.99	−1.57 [-2.60, −0.54]	*P* = 0.003	​
Lung cancer	1	5	5	-	-	-	−7.95 [-12.59, −3.30]	*P* = 0.0008	​
Prostate cancer	2	10	10	0	0	*P* = 0.96	−1.56 [-2.64, −0.49]	*P* = 0.004	​
Pancreatic cancer	1	5	5	-	-	-	−3.89 [-6.41, −1.37]	*P* = 0.002	​
Osteosarcoma	1	6	6	-	-	-	−2.10 [-3.63, −0.57]	*P* = 0.007	​
Total	8	39	39	29	9.81	*P* = 0.20	−2.05 [-2.87, −1.23]	*P* < 0.00001	​

### 3.5 The anti-tumor effects of Escin nanoparticles

Only three studies were included to investigate the effect of Escin nanoparticles on cell viability. The amalgamated findings indicated a substantial inhibitory efficacy of Escin nanoparticles on the cell viability when compared to the control group (SMD = −3.15, 95% CI = [−5.19, −1.10], *P* = 0.003; *I* ^*2*^ = 0%) (Figure 6).

**FIGURE 6:**
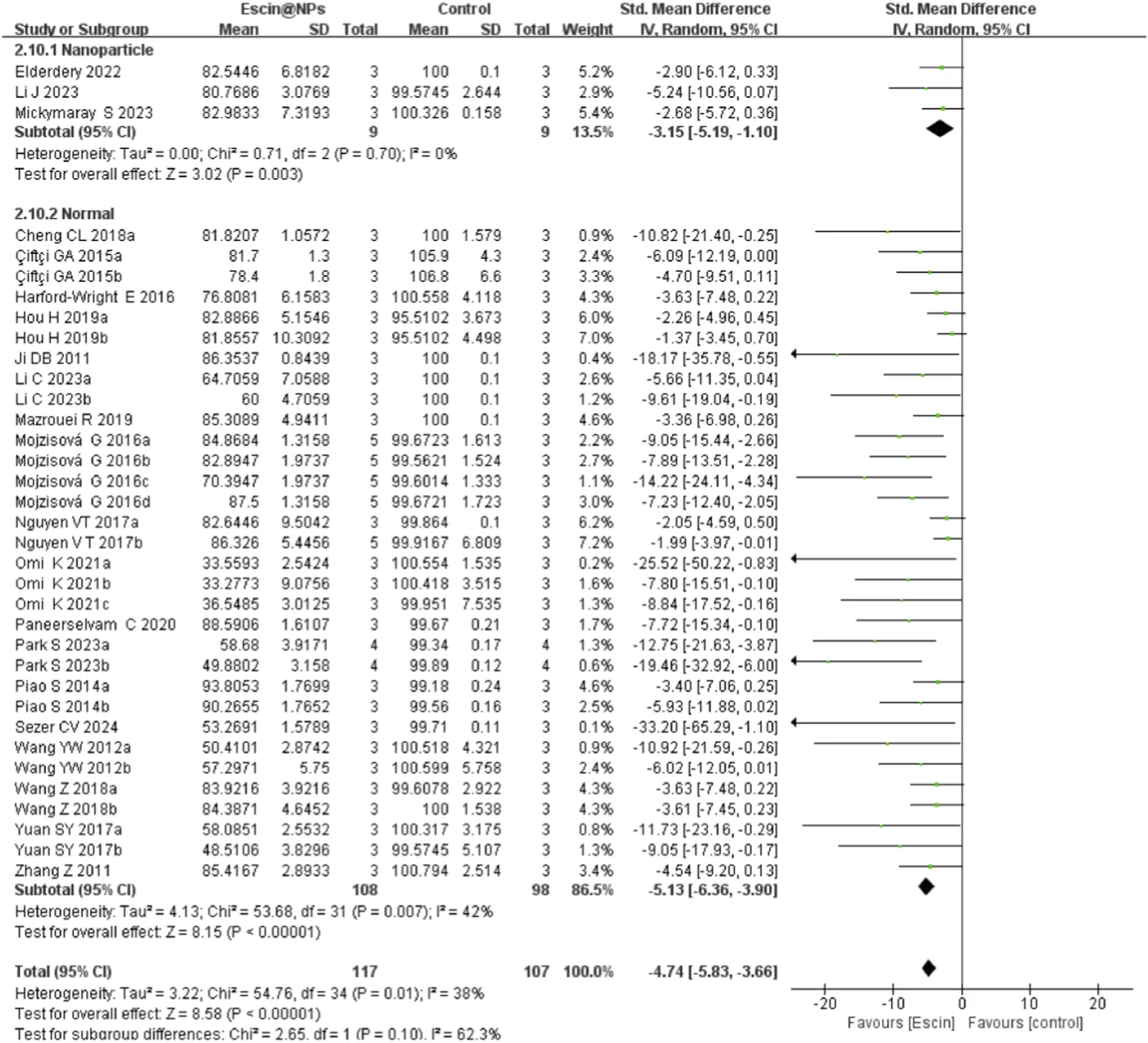
Forest plot for the effects of Escin on the cell viability of Escin-NPs compared with the free Escin. ^a, b, c, d^ was used to distinguish the different cell lines used in the same article.

### 3.6 The safety of Escin

Two articles evaluated the safety of Escin in tumor-bearing mice. Compared with the control group, Escin treatment was associated with lower ALT levels (SMD = −2.48, 95% CI = [-3.40, −1.55], *P* < 0.00001; *I* ^*2*^ = 0%) and lower AST levels (SMD = −0.98, 95% CI = [-1.69, −0.27], *P* < 0.01; *I* ^*2*^ = 0%) (Figure 7), which suggested that Escin may possess potential hepatoprotective effects.

**FIGURE 7:**
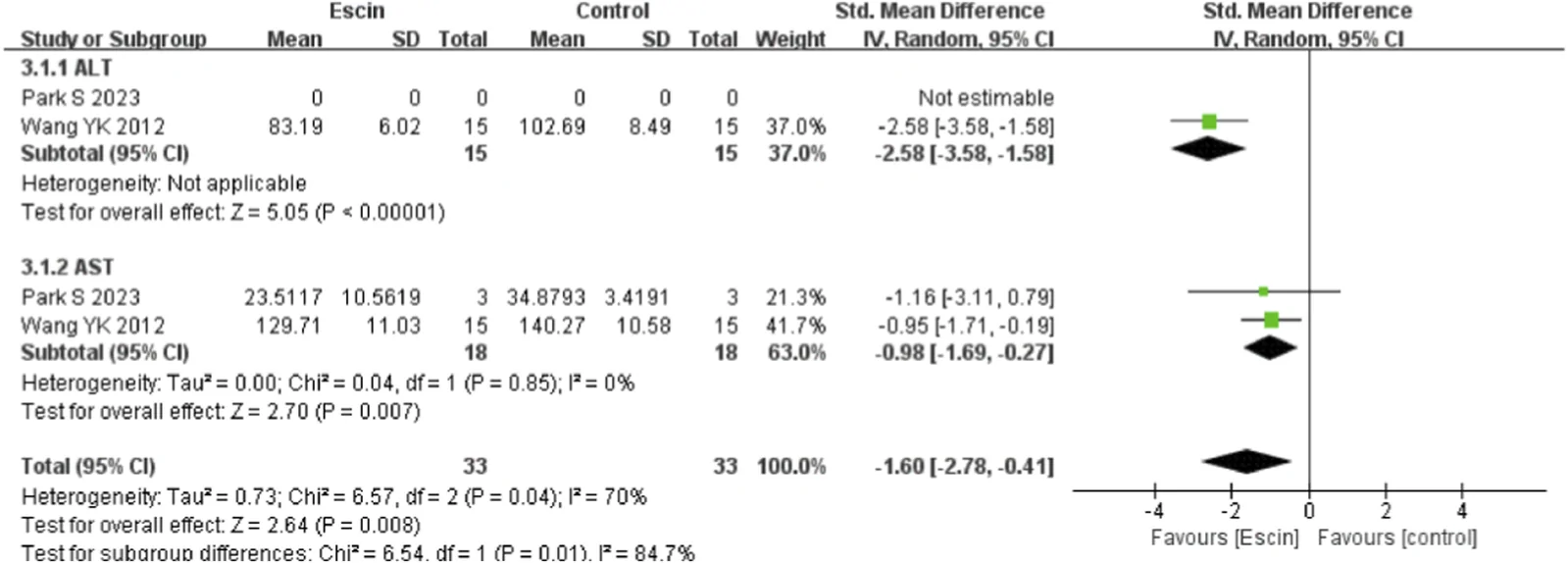
Forest plot for the effects of Escin on the liver function (ALT and AST) of rodents compared with the model control group.

### 3.7 Bias of publication

Publication bias was assessed for the indicators derived from more than eight studies. Egger’s regression test revealed significant publication bias for tumor volume (*P* < 0.01) and tumor weight (*P* < 0.01) *in vivo* (Figure 8), while cell viability (*P* < 0.001), cell apoptosis (*P* < 0.001) and cell invasion (*P* < 0.001) *in vitro* (Supplementary Figure 3). Consequently, leave-one-out sensitivity analyses were conducted for outcomes informed by at least three studies (tumor volume, tumor weight, tumor number, cell viability, apoptosis, invasion, and clone). The results showed no notable changes in any of the outcome indicators, indicating that the meta-analysis findings were relatively robust (Figure 9; Supplementary Figure 4).

**FIGURE 8:**
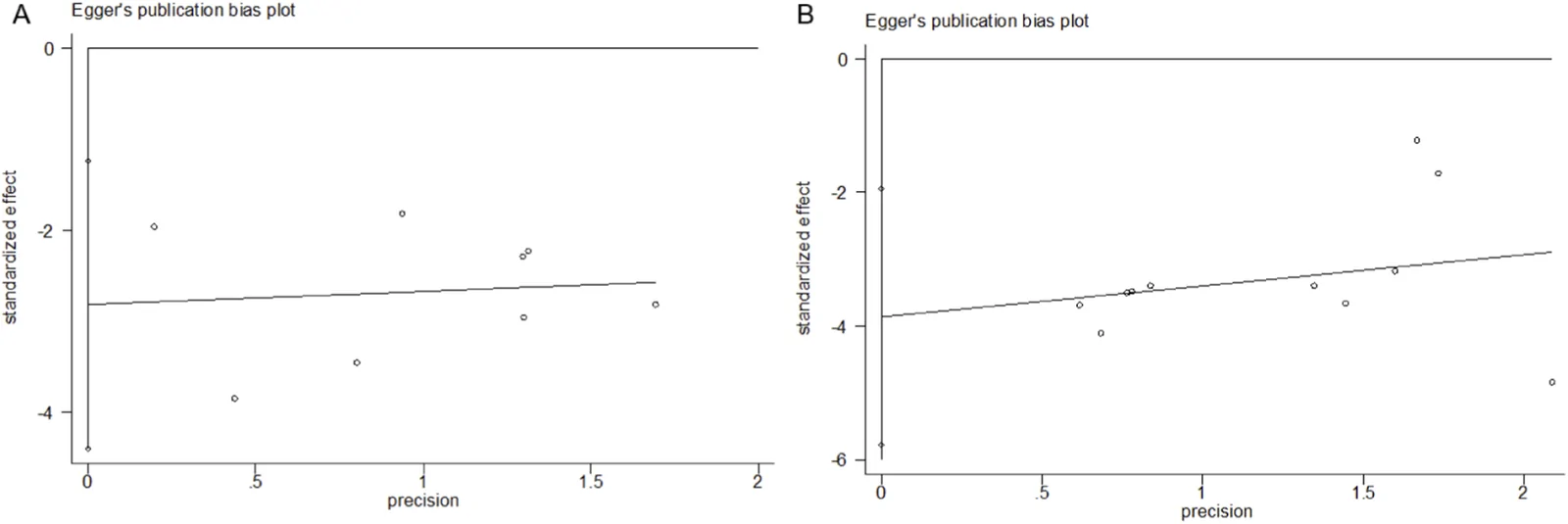
Bias of publication. **(A)** tumor volume. **(B)** tumor weight.

**FIGURE 9:**
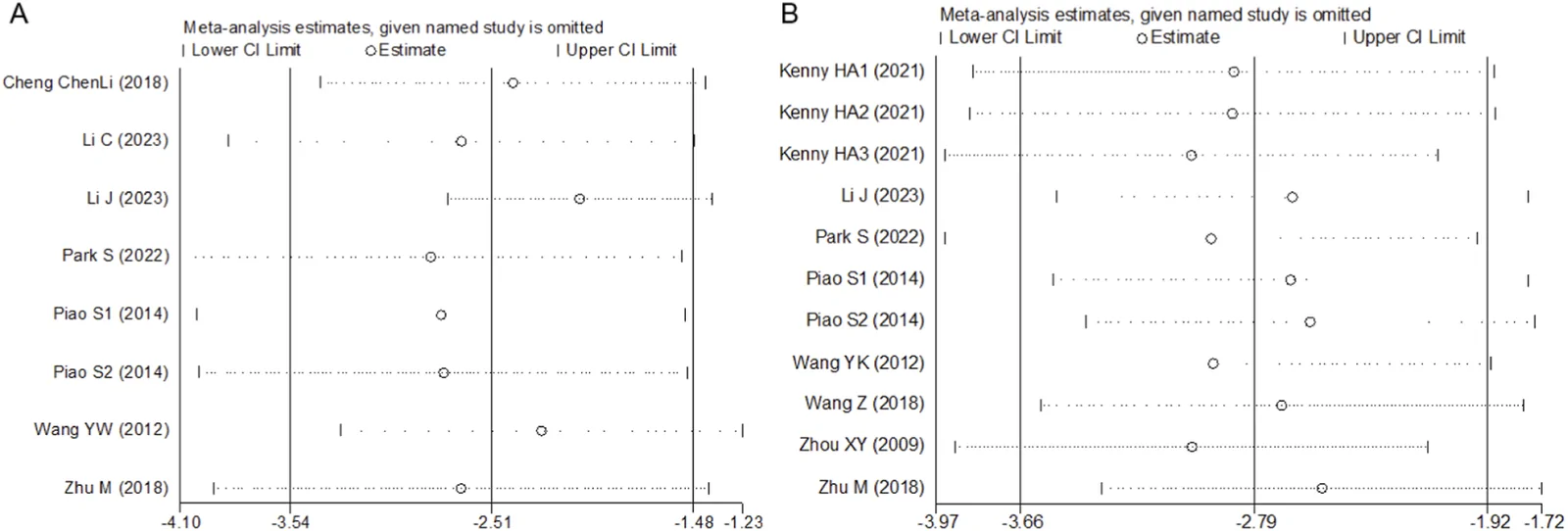
Sensitivity analyses. **(A)** tumor volume. **(B)** tumor weight.

### 3.8 Anti-cancer mechanisms

The underlying molecular mechanisms on anti-cancer effects of Escin have yet not be elucidated. Among these 30 studies detailing their mechanisms (Table 5), the most extensively investigated involved the induction of apoptosis and cell cycle arrest, particularly through ROS/NF-κB, Caspase-3 and BAX/Bcl-2 pathway. Other frequently reported mechanisms included the inhibition cell proliferation (e.g., PI3K/Akt, P21waf1/cip1), modulation of cell invasion (e.g., HIF-1α/TGF-β/MMPs, FAK/Akt), and ferroptosis (e.g., G6PD/GPX4 axis). Collectively, these findings highlighted the multi-targets of Escin in cancer.

**TABLE 5:** The mechanisms of Escin impacts the cancer therapy.

Cancer type	Anti-cancer effects and mechanisms of Escin by			Refs
	Inducing cell apoptosis	Cell proliferation	Inhibiting metastasis and invasion	
Bladder cancer	↑Caspase-3/-8/-9 ↑BAX, ↓ Bcl-2 NF-κB	G1 arrest	-	(Cheng et al., 2018)
Glioma Lung cancer	↑BAX ↑Caspase-3	G0/G1 arrest	-	(Çiftçi et al., 2015)
Leukemia/Breast cancer/Melanoma	↑ROS ↑Caspase-3,-8, -9	G1 arrest	-	(Elderdery et al., 2022; Mickymaray et al., 2023; Mojzisova et al., 2016; Park et al., 2022)
Glioblastoma	↑Sox2, Nestin	-	-	(Harford-Wright et al., 2016)
Liver cancer	↓CARMA3/NF-κB	-	-	(Hou et al., 2019)
Lung cancer	​	-	JAK2/STAT1, STAT3, P38 MAPK, NO	(Ji et al., 2011)
Melanoma	↓NF-κB	-	​	(Kwak et al., 2018)
Gastric cancer	​	-	FAK/Akt CXCL16/CXCR6	(Lee et al., 2014)
Breast cancer	↑Ferroptosis	-	-	(Li et al., 2023)
Lung cancer	↑Apoptosis	-	-	(Li et al., 2020)
Breast cancer	↓ P53/Bcl2	-	-	(Mazrouei et al., 2019)
Pancreatic cancer	↓NF-κB	-	IL-8/VEGF	(Omi et al., 2021)
Lung cancer/Melanoma	↑ROS	-	HIF-1α/TGF-β/MMPs	( Paneerselvam and Ganapasam, 2020; Vejselova Sezer, 2024 )
Colon cancer	-	↓P21waf1/cip1 G0/G1 arrest	-	(Patiolla et al., 2006)
Prostate cancer	-	G1 and G2/M arrest	-	(Piao et al., 2014)
Pancreatic cancer	↓P65/IκBα/p-IκBα	-	-	(Rimmon et al., 2013)
Liver cancer	↓Bcl-2, Bcl-xL	↓Cyclin D1	-	(Tan et al., 2010)
Breast cancer	-	-	↓LOXL2	(Wang et al., 2016)
Liver/Kidney cancer	↑ROS	-	-	(Wang et al., 2012a; Yuan et al., 2017)
Pancreatic cancer	↓NF-κB/IκB	G0/G1 arrest	-	(Wang et al., 2012b)
Colorectal cancer	↑P62/ATM/γH2AX	-	-	(Wang et al., 2018)
Leukemia	-	G0/G1 arrest	-	(Zhang et al., 2011)
Liver cancer	↑Caspase-3	G1 arrest	-	(Zhou et al., 2009)
Osteosarcoma	Through ROS/P38 MAPK	-	-	(Zhu et al., 2017)
Osteosarcoma	-	↓PI3K/Akt	-	(Zhu et al., 2018)

## 4 Discussion

Escin is clinically used for venous insufficiency, pain, inflammation, and edema (Marciano et al., 2024). This present study systematically synthesized and quantitatively evaluated preclinical evidence on the anti-tumor effects of Escin and its nanoparticles. The results indicated that Escin and its nanoparticles exhibited potential anti-tumor effects, as evidenced by reduced cell viability, induced cell-cycle arrest and apoptosis, inhibited cell invasion and migration *in vitro*, and decreased tumor volume and weight in mouse models. Furthermore, it was suggested that Escin may had potential protective effects against liver injury in tumor-bearing mice. However, given the limited number of included studies, these findings should be interpreted cautiously. To our knowledge, this is the first systematic review and meta-analysis to specifically evaluate the therapeutic potential of Escin and its nanoparticle formulations in cancer. Previous related literature consisted primarily of narrative reviews, such as those by Fazliev et al. (Fazliev et al., 2023) and Cheong et al. (Cheong et al., 2018), which offered qualitative summaries without quantitative synthesis. Our findings were broadly consistent with the conclusions of Fazliev et al. (Fazliev et al., 2023), who reported that Escin exerted anti-cancer effects through apoptosis induction, cell-cycle arrest, and inhibition of metastasis. However, our meta-analysis extended this work by providing pooled quantitative effect estimates and by systematically assessing nanoparticle formulations as a distinct therapeutic strategy. Collectively, these findings provided quantitative preclinical evidence supporting Escin as a promising therapeutic strategy for cancer treatment.

Substantial heterogeneity was observed among the included studies, which was common in preclinical meta-analyses due to variations in experimental models and protocols. Subgroup analyses suggested that animal strains, gender, animal numbers, and type of tumor were important contributors on tumor weight (Moreno et al., 2018). Specifically, a wide range of animal strains and models with different immune status were employed, including immunocompetent mice (e.g., BALB/c, C57BL/6) and immunodeficient strains (e.g., BALB/c nude and HSD nude mice). The subgroup analysis indicated that significant difference was found in immune-deficient mice (such as BALB/c nude, HSD nude), but not in immune-healthy mice. This was maybe likely that only three studies involved immune-healthy mice, and the animal strains in each study were different. In addition, the genetic background differences among different inbred mouse strains can affect the activity of drug-metabolizing enzymes and the growth rate of tumors (Malkinson, 1991), thereby amplifying heterogeneity. This suggested that future research should prioritize models with high clinical convertibility and sound immunity, and clearly define the source and genetic background of the reported strains. Secondly, the gender of experimental animals was another source of heterogeneity that cannot be ignored (Cheng, 2006). The results showed that there were differences in the responses to Escin between male and female animals, which may be related to the regulatory effect of sex hormones on drug-metabolizing enzymes (such as CYP450), the interference of estrogen on tumor proliferation signaling pathways, and the differences in immune responses due to sex dimorphism (Zanger and Schwab, 2013). Thirdly, the effect size of Escin varied significantly among different transplant tumor models such as ovarian cancer, lung cancer and breast cancer. This is attributed to the inherent differences in drug sensitivity of different tumor cells, the activation threshold of apoptotic pathways, and the tumor microenvironment (such as vascular density and hypoxia status) (Yang et al., 2024). Additionally, a striking finding emerged from the subgroup analysis based on group size. Heterogeneity was significant among studies using >5 animals per group (*P* = 0.03), but not among those with ≤5 animals per group (*P* = 0.87). It also should point out that route of administration was an important source of heterogeneity. The i. v. administration achieved nearly 100% bioavailability and rapid systemic exposure. The i. p. administration permited fast absorption and high bioavailability, with minor variability. In contrast, oral/i. g. resulted in low bioavailability due to gastrointestinal degradation and extensive first-pass metabolism. These pharmacokinetic differences could, in principle, translate into different therapeutic effects. In our subgroup analysis, the i. v. route showed the largest numerical reduction in tumor weight, which was consistent with its higher systemic exposure. However, the difference among routes did not reach statistical significance. This may be explained by several factors such as dose regimens and limited number of included studies. In summary, the substantial heterogeneity observed in this meta-analysis was not attributable to a single variable, but rather reflected the combined influence of multiple interacting experimental factors. Subgroup analysis identified the primary moderators; however, the limited number of studies within certain subgroups reduced statistical power, warranting cautious interpretation of these findings (Festing and Altman, 2002). Standardizing animal models, dose regimens, formulation characterization, and outcome assessments is critical to enhance the reliability and translational relevance of preclinical evidence on Escin for cancer therapy.

Free Escin has some limitations that hinder its clinical application, such as poor water solubility, low bioavailability, and lack of tumor-targeting ability (Wang et al., 2022). These drawbacks severely constrain its translational potential as an anti-cancer agent. Nano-based drug delivery systems offer a feasible strategy to overcome these bottlenecks. To date, multiple Escin-loaded nanoformulations have been developed, encompassing polymer nanofibers, solid lipid nanoparticles, and inorganic nanocomposites (Elderdery et al., 2022; Escobedo et al., 2024). Among them, an intelligent pH-responsive cellulose nanofibers-Escin nanoformulation was established with the cumulative release rate reaches 85% within 72 h in the simulated tumor microenvironment (pH 5.0), while only 35% was released at the physiological pH of 7.4, achieving precise delivery of ‘on-demand drug release’ (Seth et al., 2026). Meanwhile, an Escin-loaded glutaraldehyde-albumin nanoparticle system was found to enhance anti-cancer activity on lung cancer A549 cells through activiting oxidative stress (Munusamy et al., 2025). In this study, a significant reduction of cell viability was observed in Escin nanoparticle-treated group compared with the untreated control group. Regrettably, a direct comparison between Escin nanoparticles and free Escin could not be performed owing to the limited number of relevant studies available. Nevertheless, this observation points to an important direction for subsequent investigations, as direct comparison will be essential to fully validate the translational benefits of Escin nanoformulations.

Multiple anti-tumor mechanisms have been clarified for Escin (Yan et al., 2025). Among the most extensively studied pathways were the induction of apoptosis and cell cycle arrest, as well as the inhibition of cell proliferation, migration, and invasion. The underlying mechanisms seemed to be mediated primarily through the NF-κB signaling pathway, while ROS and JAK/STAT signaling also played contributory roles. Ferroptosis, an iron-dependent non-apoptotic cell death process (Dixon and Olzmann, 2024), had additionally been proposed as a possible mechanism. Other mechanisms included FAK/Akt, which intensively correlated with tumor occurrence, drug resistance and regulation of bone homeostasis (Katoh, 2024), as well as modulation of the tumor microenvironment, particularly through extracellular matrix remodeling (Winkler et al., 2020). Oxidative stress also played a role in the anti-tumor action of Escin (Hussain et al., 2024). It had been shown to reduce cell ROS levels, incorporate MDA, GSH, SOD and LDH, thereby inducing cancer cell apoptosis. Overall, although apoptosis remains the primary mechanism, future research should explore other forms of regulated cell death, including mitoptosis, autophagy, and cuproptosis, as key research directions.

There were also shortcomings in this meta-analysis. Firstly, the quality of the literature reviewed was not optimal. Some studies failed to detail their randomization process, while the others only mentioned the term ‘randomization’ without elaborating. Therefore, methodological quality issues included in the study might affect the evidence strength of this meta-analysis. Secondly, the use of data extraction software instead of analyzing raw data, and the lack of information, not provided by the authors, such as the sex or weight of the animals, posed challenges for a robust meta-analysis. Thirdly, the heterogeneity observed in the study may be attributed to the multiple factors such as sample size, cancer types. Fourthly, some cancer-specific subgroups contained very few studies, which may reduce the reliability of the subgroup estimates. Therefore, the observed differences across cancer types should be considered exploratory and require confirmation in future studies with larger sample sizes. Additionally, the majority of existing studies were characterized by their short-term duration and small sample sizes.

## 5 Conclusion

This systematic review and meta-analysis synthesized existing preclinical evidence and provided the first quantitative assessment of the efficacy, safety, and underlying mechanisms of Escin and its nanoparticles in cancer therapy. At the cellular level, both Escin and its nanoparticles reduced cell viability. Escin also induced cell apoptosis and cell-cycle arrest, and inhibited cell migration and invasion. In animal cancer models, Escin significantly decreased tumor volume, tumor weight, and tumor number compared with controls. Mechanistically, the anti-tumor effects appeared to operate particularly through the NF-κB signaling pathway, with ROS and JAK/STAT signaling also playing contributory roles. In addition, Escin may have potential hepatoprotective effects in tumor-bearing mice; however, this preliminary observation requires validation in larger, well-designed studies. Despite these promising findings, the reliability of the pooled estimates may be affected by the quality and quantity of the included studies and by substantial heterogeneity across experimental models and protocols. Therefore, larger, well-designed preclinical studies are warranted to confirm these effects, clarify the comparative advantages of nanoformulations, and support future clinical translation.

## Data Availability

The original contributions presented in the study are included in the article/Supplementary Material, further inquiries can be directed to the corresponding author.
