# Supplementary material for: Therapeutic potential of escin and its nanoparticle formulations in cancer -a systematic review and meta-analysis of preclinical study
Source: Front Pharmacol. 2026 Oct 30;17:1988783. doi: 10.3389/fphar.2026.1988783 (PMC13759835; doi:10.3389/fphar.2026.1988783)
Supplement: Supplementary file 1 [file DataSheet1.doc]

**Supplementary Figures and Tables**

**Supplementary Fig. 1.** **The risk of bias graph of included studies using SYRCLE’s risk of bias tool for animal studies**.

A

B

**Supplementary Fig. 2.** **Forest plot for the anti-tumor effects of Escin on Rodents compared with the model control group. (A) cell clone, (B) tumor volume, (C) cell adhesion and cell proliferation.** **^a, b, c^ was used to distinguish the different tumor types used in the same article.**

A B

C D

E

**Supplementary Fig. 3. Bias of publication. (A) Cell viability, (B) Cell apoptosis, and (C) Cell invasion, (D) Cell clone, (E)Tumor number**

A B

C D

E

**Supplementary Fig. 4. Sensitivity analyses. (A) Cell viability, (B) Cell apoptosis, and (C) Cell invasion, (D) Cell clone, (E)Tumor number.**

**Supplementary Table 1 The characteristics of Escin nanoparticle formulations**

| **Nanoparticle**  **[size (nm)/Zeta (mV)PDI/Drug loading%]** | **Composition** | **Treatment dose** | **Ref** |
| --- | --- | --- | --- |
| 116 /0.283/-/- | CuO-TiO2-Chitosan-Escin | 1,1.5,2 mg/mL | Elderdery 2022 |
| 148.9/−30/ 0.205/50% | PTX-A and FKA-A | 10 μg/kg | Li J 2020 |
| 101/-/-/- | MgO-Chitosan-Pluronic F127-Escin | 10 µg/mL | Mazrouei 2019 |
